# A High Visceral-to-Skeletal Muscle Area Ratio on Cross-Sectional Imaging Is Associated With Failure of Standard Ustekinumab Doses: A Multicenter Study

**DOI:** 10.14309/ctg.0000000000000722

**Published:** 2024-06-01

**Authors:** Zhi Tan, Andrew Chin, Christopher J. Welman, Lena Thin

**Affiliations:** 1 Department of Gastroenterology, Fiona Stanley Hospital, Murdoch, Australia;; 2 Department of Gastroenterology, Royal Perth Hospital, Perth, Australia;; 3 Department of Radiology, Royal Perth Hospital, Perth, Australia;; 4 Department of Imaging, Fiona Stanley Hospital, Murdoch, Australia;; 5 Department of Internal Medicine, UWA Medical School, Perth, Australia.

**Keywords:** Crohn's disease, body mass index, adiposity

## Abstract

**INTRODUCTION:**

Anti-interleukin 12/23 agents have shown greater durability in response compared with anti-tumor necrosis factor α agents. Data on the association between body composition (BC) or body mass index (BMI) and ustekinumab's therapeutic response is limited. We aimed to evaluate the impact of BC on time to failing standard doses of ustekinumab in patients with Crohn's disease (CD).

**METHOD:**

Patients with CD aged 16 years and older from 2 tertiary centers were studied retrospectively. Included patients had abdominal imaging within 6 months of ustekinumab induction and were followed until April 30, 2022. An experienced abdominal radiologist blinded to the clinical information measured the area of visceral fat area and skeletal muscle area at the mid L3 vertebral level, with values corrected for height^2^ to derive respective indices (visceral fat index [VFI], skeletal muscle index [SMI]) and the VFI:SMI ratio.

**RESULTS:**

Ninety-nine patients met inclusion criteria. The mean age at ustekinumab induction was 46.6 (±1.6) years. The median BMI (interquartile range) was 26.5 (22.6–30.8). Twenty-four patients (24.2%) did not respond or lost response to standard doses of ustekinumab over the follow-up duration. A younger age (hazard ratio 0.96, 95% confidence interval 0.94–0.99, *P* = 0.01) and a VFI:SMI ratio >1.6 (hazard ratio 4.65, 95% confidence interval 1.73–12.45, *P* = 0.002) were both associated with a shorter time to failing ustekinumab at standard doses on multivariate analysis. BMI, notably, had no association with the primary outcome.

**DISCUSSION:**

A high VFI:SMI ratio is associated with an increased risk of failing standard doses of ustekinumab. BC measurements derived from cross-sectional imaging at the start of ustekinumab therapy is a useful indicator for therapeutic durability.

## INTRODUCTION

The prevalence of inflammatory bowel disease (IBD) is increasing worldwide, with up to 7 million people affected globally with this chronic relapsing and remitting disease (1). Advance therapies with biologics, and more recently small molecules, have become the mainstay for managing moderate to severe IBD. Several risk factors have been identified to affect biologic loss of response (LOR), and of these, there has been particular interest in the role of body mass index (BMI), adipose volume, and visceral adiposity. A high adipose burden, particularly visceral adiposity, has been associated with lower remission rates (^2,3^) and an increased requirement for biologic dose escalation in obese patients with IBD.

Like IBD, the worldwide prevalence of obesity is increasing. Forty percent and 15%–40% of patients with IBD are, by definition, overweight and obese, respectively (4,5). Obesity in itself is a chronic inflammatory condition with elevated levels of circulating inflammatory cytokines and adipokines, creating a biologic sink for anticytokine therapies that treat IBD. A recent systemic review has shown higher visceral adipose tissue (VAT) volume to be associated with a high prevalence of complicated Crohn's disease (CD) phenotypes, in contrast with lean muscle mass, which seems to be associated with a lower risk of complicated IBD (6).

The role of BMI in optimizing anti-tumor necrosis factor (TNF) α dosing has been contentious. BMI is independently associated with LOR in adalimumab-treated patients, where doses are fixed and not weight-based, unlike infliximab (7–9). Our previous work showed that while BMI did not associate with infliximab LOR, but did for adalimumab LOR, it was the visceral fat index (VFI; visceral fat area at L3 level corrected for stature) and ratio of visceral fat to skeletal muscle area that correlated well with adalimumab LOR and lower infliximab drug levels. Subsequent studies have also corroborated with our results confirming that it is the VAT volume rather than BMI that affect biologic resistance (2,3).

Ustekinumab, an interleukin-12/23 anti-p40 agent, has been approved in many countries for the treatment of moderate to severe CD and ulcerative colitis (UC). A *post hoc* analysis of the UNITI 1 and 2 and IM-UNITI studies showed no effect of BMI on clinical efficacy (clinical remission and steroid-free clinical remission) despite being independently and inversely associated with ustekinumab drug levels (10). As a non-weight-based agent for maintenance, it is conceivable ustekinumab may behave in a similar fashion to adalimumab, having a limited efficacy in those with high BMI at standard doses, despite the lack of current evidence showing this. Dose escalation with or without dose reinduction is effective in recapturing response in those experiencing ustekinumab LOR (11). It is unknown, however, which patients, and what clinical characteristics, are associated with the highest risk of ustekinumab LOR. Based on our previous work and those of others (2,3,9), we hypothesize that patients with a high visceral adipose burden would lose response to standard doses of ustekinumab faster and represent a group of patients that may need ustekinumab dose intensification earlier and/or careful attention to weight management. In this retrospective cohort study, we sought to investigate the relationship between body composition (BC) and (i) the risk of failing to induce or maintain ustekinumab response at standard doses over time and (ii) the clinical parameters that may be associated with failing ustekinumab at standard doses.

## METHODS

### Study design

This study was a retrospective, multicenter, cohort analysis across 2 tertiary academic hospitals in Western Australia with specialist IBD clinics.

### Outcomes

The primary outcome measured was the prevalence of ustekinumab failure to standard doses for induction and maintenance therapy. The secondary outcome measured was the time to failing standard ustekinumab doses, as defined below, across different BCs.

### Subject inclusion criteria

**Figure 1.**
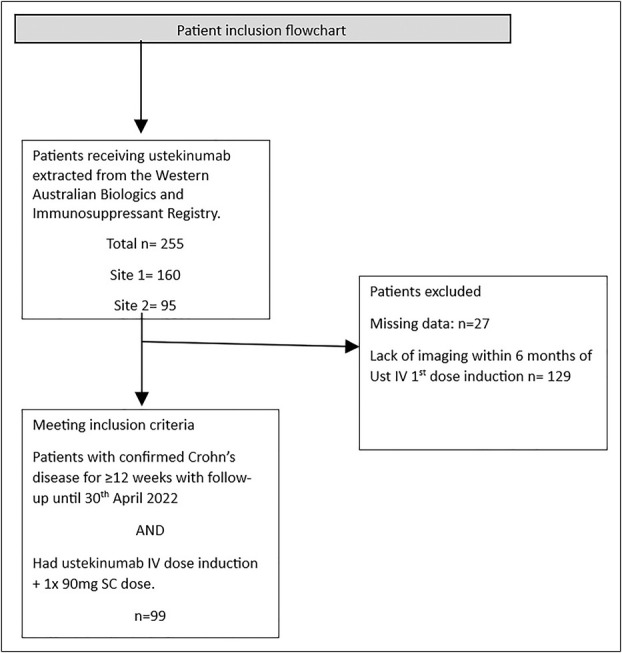
Flowchart of patients meeting inclusion and exclusion criteria. IV, intravenous; SC, subcutaneous.

Patients 16 years and older with a confirmed diagnosis of CD by standard clinical, endoscopic, histopathological, and radiological criteria (12) for at least 3 months were eligible for inclusion. Data were extracted from a prospectively maintained statewide IBD database (The West Australian Biologics and Immunosuppressant Registry) and supplemented with details from electronic medical records. Included patients must have had either an abdominal computed tomography (CT) scan or magnetic resonance imaging (MRI) scan within 6 months of their first intravenous (IV) ustekinumab induction date (Figure 1).

### Definitions

We defined the primary outcome of ustekinumab failure as a patient experiencing an inadequate response (IR) or LOR to standard ustekinumab induction and maintenance doses. We defined an IR to ustekinumab as no change in CD Activity Index (CDAI) after the first IV dose of ustekinumab and assessed anytime in the first 8 weeks from the first dose of IV ustekinumab. We defined LOR to ustekinumab as an initial response with a decrease of at least 100 points in CDAI, followed by LOR (any increase in CDAI thereafter) after at least 12 weeks of therapy with ustekinumab, which allowed for at least 1 IV induction dose and 1 subcutaneous (SC) dose. An IR or LOR was accompanied by at least 1 objective evidence of disease activity that had not improved from baseline, acquired within a month of the clinical assessment; including presence of ulceration at endoscopy, and/or presence of active disease on MRI/CT, and/or raised fecal calprotectin >250 μg/g, and/or raised C-reactive protein (CRP) >10 mg/L; leading to either (i) a change in biologic agent, (ii) surgical resection, or (iii) dose intensification (ustekinumab dose interval shortening with or without IV dose reinduction and/or addition of immunomodulator). If the treating IBD physician felt the patient was at risk of progressive disease or complications, and had failed multiple prior therapies, assessments for a prompt response occurred earlier than 8 weeks and a second dose of IV ustekinumab or the first SC dose at week 3–4 was given if they had not responded.

Patients met criteria for the primary end point if they fulfilled the criteria for ustekinumab failure as defined above. Patients were followed up from the time of the initial IV induction ustekinumab dose until patients met the primary end point. Patients that were lost to follow-up, were noncompliant with therapy or died, or were censored at the date of the last available clinic follow-up or death. The remaining patients were followed until the closing study date of April 30, 2022.

### Data collection

Data extraction began from January 1, 2017. Extracted data variables included patient demographics such as sex, age, smoking status (ex, never, current) and BMI. CD-specific data extracted included the date of diagnosis, disease duration, Montreal classification, IBD therapy including use of immunomodulators or steroids at ustekinumab induction, and CD-related surgery and disease activity score (CDAI) at induction. Serological data such as CRP (mg/L), albumin (g/L), hemoglobin (g/L), platelet count (×10^9^), and fecal calprotectin (µg/g) within 3 months of the cross-sectional imaging were recorded. Visceral fat area (VFA), SC fat area (SFA), and skeletal muscle area in centimeter squared (cm^2^), SFA and abdominal circumference (AC) in centimeters were measured from CT and MRI at the L3 vertebral levels (see below). Values were normalized for height (m^2^) and expressed as an index (e.g., VFA/height^2^ = VFI).

### Imaging acquisition and BC measurement

The method used for image acquisition and BC measurement in our current study was the same method used in our previously published research (9). A single radiologist (C.J.W.) blinded to the clinical data reviewed all study images obtained using a range of multislice CT and 1.5 T MRI scanners from different manufacturers according to standard protocols for imaging CD. Subject images were retrieved in Digital Imaging and Communications in Medicine format. For CT, the 3 mm axial slice was cross-referenced with the sagittal reformatted images with the morphologic L5/S1 junction as reference. For MRI, the axial slice was cross-referenced with the coronal half-Fourier acquisition single-shot turbo spin echo or volumetric interpolated breath-hold examination sequences. The slice was obtained from the middle of the L3 vertebral body, unless there was significant artifact at that level, in which case the first slice immediately cranial or caudal to this level was chosen. Half-Fourier acquisition single-shot turbo spin echo, true fast imaging with steady state precession, and volumetric interpolated breath-hold examination images were assessed.

BC was performed using the National Institutes of Health ImageJ software (version 1.52a) for MRI and Sliceomatic (version 5.0 Rev 12; TomoVision, Magog, Canada) using the ABACS mode (Voronoi Health Analytics, Coquitlam, Canada) for CT. For CT images, the Hounsfield unit value ranges were used to differentiate between the fat and muscle components based on tissue-specific attenuation values for skeletal muscle (−29, +150) and adipose tissue (−190, −30). The color-coded overlay was assessed for correct segmentation; any errors were manually corrected using Edit mode following standard anatomic boundaries. For MRI images, visual identification of tissue planes allowed manual segmentation using the ImageJ free-hand tool with a Microsoft Surface Pen on a Microsoft Surface Pro 4. A previous analysis of BC assessments using CT and MRI measurements have shown to produce similar results (13).

The AC measurement excluded stoma bags. Where the body edge was excluded from the field of view, no attempt was made to extrapolate missing tissue, potentially underestimating AC and SFA. VFA excluded large mesenteric vessels, mesenteric nodes, bowel loops, and solid organs. In cases where there was an abdominal wall hernia or stoma site, the herniated fat was included in the VFA and excluded from the SFA measurements.

### Statistical considerations

The patients' baseline demographics and characteristics were analyzed using descriptive statistics. Normally distributed continuous variables were expressed as mean and SD while nonparametric data were expressed as median and interquartile range (IQR). Categorical variables were expressed as proportions. A Kaplan-Meier survival analysis was performed to determine the median time to ustekinumab failure at standard doses between the different BC groups. The patient's follow-up was censored at the date of their last available clinic appointment or April 30, 2022, whichever was earlier.

We used univariate and multivariate Cox regression analyses to determine factors that were associated with the risk of ustekinumab failure at standard doses over the follow-up period. Covariates included in the final multivariate model included any factor identified on the univariate analysis to be associated with the primary end point with a *P* value <0.2. Covariates of interest included a priori in the univariate analysis were age, sex, albumin level, CRP level, BMI, disease duration, prior biologic failure status, concurrent immunomodulator use, presence of perianal disease, and smoking status (current vs none/ex-smoking). A backward stepwise regression method was used, and covariates with a *P*-value >0.05 were removed at each step, leaving only the significant variables that affected the model at the end. SPSS version 20 was used to perform the statistical analyses with a 2-sided *P*-value <0.05 deemed statistically significant.

### Ethical consideration

This study was approved by the institution's low risk ethics (GEKO) committee, reference number: GEKO 47857. Individual patient consent was not required as data were analyzed in a deidentified manner with patient identifying details stored securely in password-encrypted files, and reidentification was not permitted.

## RESULTS

In total, 99 patients met inclusion criteria, with 50.5% (50/99) female patients. The mean age of the cohort was 46.6 years (±1.6). The median BMI was 26.5 kg/m^2^ (IQR 22.6–30.8); however, most of the cohort (78/99, 78.8%) did not have a documented metabolic risk factor. Thirty-six of 99 patients (36.4%) were naïve to prior biologic exposure, defined as a prior IR or intolerance to the biologic. The median duration of disease at the time of ustekinumab induction was 135 months (IQR 43–223). Patients who commenced ustekinumab had a moderate to severe disease activity with a median CDAI score of 313 (IQR 110.5–343) at induction. Seventy-one of 99 patients (71.7%) had no concurrent immunomodulator treatment of at least 6 months duration. The median BC measurements for the cohort and other baseline characteristics are summarized in Table 1.

**Table 1.** Patient baseline characteristics

Characteristic	Total (n = 99)
Male, n (%)	49 (49.5)
Age, yr, mean (±SE)	46.6 (1.6)
Smoking status	
Ex-smoker	54 (54.5)
Current	20 (20.2)
Never	25 (25.3)
BMI, median (IQR)	26.5 (22.6–30.8)
Metabolic risk factors, n (%)	
None	78 (78.8)
Hypertension	4 (4)
Diabetes	1 (1)
Dyslipidemia	3 (3)
Ischemic heart disease	2 (2)
Fatty liver	4 (4)
Multiple (2 or more)	7 (7.1)
Extraintestinal manifestations at the time of induction, n (%)	
None	90 (90.9)
Psoriasis	5 (5.1)
Erythema nodosum	1 (1)
Primary sclerosing cholangitis	1 (1)
Enteropathic arthritis	1 (1)
Pyoderma gangrenosum	1 (1)
Previous biologic failures, n (%)	
Bio-naïve	36 (36.4)
Infliximab	6 (6.1)
Adalimumab	23 (23.2)
Vedolizumab	4 (4)
Multiple (2 or more)	30 (30.3)
Age at onset, n (%)	
≤16 yr (A1)	7 (7.1)
17–40 yr (A2)	72 (72.7)
>40 yr (A3)	20 (20.2)
Disease location, n (%)	
Ileal (L1)	39 (39.4)
Colonic (L2)	13 (13.1)
Ileocolonic (L3)	47 (47.5)
Upper GI involvement (L4)	5 (5.1)
Disease behaviour, n (%)	
Inflammatory (B1)	36 (36.4)
Stricturing (B2)	43 (43.4)
Penetrating (B3)	20 (20.2)
Perianal disease modifier (P), yes, n (%)	17 (17.2)
Disease duration at ustekinumab induction, mo, median (IQR)	135 (43–223)
Duration of patient follow-up, wk, median (IQR)	62 (31–101)
CDAI score at ustekinumab induction, median (IQR)	313 (110.5–343)
Values at baseline before ustekinumab induction, median (IQR)	
CRP (mg/L)	19.2 (4.8–63)
Albumin (g/L)	39 (34–42)
Hemoglobin (g/L)	129 (112–137)
FC (µg/g)	305 (142–977)
No. of prior intestinal resections	
0	39 (39.4)
1	36 (36.4)
>1	24 (24.2)
Any corticosteroid (budesonide or prednisolone) within 6 mo of ustekinumab induction	46 (46.5)
Concurrent immunomodulator for >6 mo with ustekinumab, n (%)	
None	71 (71.7)
Methotrexate	12 (12.1)
Thiopurines	16 (16.2)
Ustekinumab dose interval shortening (with or without IV dose re-induction) to	
4-weekly	18 (18.2)
6-weekly	2 (2)
Remained on 8-weekly	78 (78.8)
Body composition domains	
Visceral fat index (cm^2^/m^2^), median (IQR)	41 (18.3–63.3)
Skeletal muscle index (cm^2^/m^2^), median (IQR)	45.1 (38.8–54.5)
Abdominal circumference (cm), mean (±SE)	96.2 (±1.9)
VFA:SMA ratio, median (IQR)	0.88 (0.5–1.3)

BMI, body mass index; CDAI, Crohn's Disease Activity Index; CRP, C-reactive protein; FC, fecal calprotectin; GI, gastrointestinal; IQR, interquartile range; IV, intravenous; SE, standard error; SMA, skeletal muscle area; VFA, visceral fat area.

**Figure 2.**
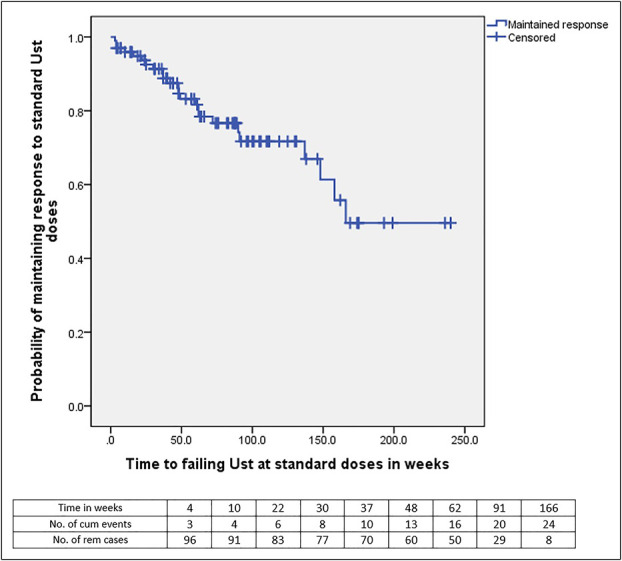
Kaplan-Meier survival analysis of ustekinumab-treated cohort. Kaplan-Meier survival analysis of failing ustekinumab at standard doses of the whole cohort (n = 99) as defined as rise in Crohn's Disease Activity Index with an objective marker of disease activity (C-reactive protein, fecal calprotectin, endoscopy, radiology) plus receiving (**a**) ustekinumab dose interval shortening with or without intravenous ustekinumab reinduction, (**b**) surgical resection, or (**c**) change in biologic agent. Ust, ustekinumab.

The proportion of patients who met primary end point criteria by the census date of April 30, 2022, is summarized in Table 2, and the Kaplan-Meier survival analysis on the probability of maintaining response to ustekinumab, as defined by the primary end point criteria, is depicted in Figure 2. The median duration of follow-up for the cohort was 62 weeks (IQR 31–101). Over this period of follow-up, 75 of 99 patients (75.8%) maintained a response to standard ustekinumab doses and 24 patients (24.2%) did not respond or lost response to standard doses of ustekinumab (3 [3%] experienced IR and 21 [21.2%] experienced LOR as per our a priori definitions). No patients changed to a different biologic as ustekinumab was the most recently reimbursable biologic added to the Australian Pharmaceutical Benefits Scheme at the time of data collection, and patients on this agent had usually failed multiple biologics. Of the 24 patients who met primary end point criteria, 4 (4%) went on to have an intestinal resection and 20 (20.2%) had ustekinumab dose intensification with either interval shortening and/or required an IV dose reinduction. The median time to failing ustekinumab at standard doses in the cohort was 166 weeks (95% confidence interval [CI] 142.5–190.3) (Figure 2).

**Table 2.** Proportion of patients meeting primary outcome by criteria

Primary outcome: Percent meeting LOR criteria by the end of follow-up									
Outcome		Objective LOR criteria							Total n, % within outcome
		No objective criteria	Raised CRP	Raised FC	Raised CRP and FC	Endoscopic activity	Radiological activity	All: raised CRP, FC, endoscopic or radiological activity	
Not meeting primary end point criteria	Count % within LOR criteria	75	0	0	0	0	0	0	75, 75.8%
Surgical resection	Count % within LOR criteria	0	1	1	0	2	0	0	4, 4%
Ustekinumab IV dose reinduction and/or interval shortening	Count % within LOR criteria	0	3	1	1	4	7	4	20, 20.2%
Total	Total n, % within LOR criteria	75, 100.0%	4, 100.0%	2, 100.0%	1, 100.0%	6, 100.0%	7, 100.0%	4, 100.0%	99, 100.0%

The proportion of patients meeting primary end point criteria. Patients with an inadequate response within the first 8 weeks, or LOR, defined as an initial drop of >100 points on CDAI followed by an increase in CDAI, with any objective criteria of active disease that is not improved from baseline (CRP and/or FC and/or endoscopic activity and/or radiological activity after the first 12 weeks of therapy) AND either (i) change of biologic (ii) requiring intestinal resection or (iii) ustekinumab dose interval shortening with or without reinduction. No patients in this cohort changed biologic, thus this group was excluded from the table. Endoscopic activity: presence of any mucosal ulceration. Radiological activity: presence of any bowel wall thickening on CT or MRI and/or mesenteric stranding and/or mural enhancement on CT or MRI scan.

CDAI, Crohn's Disease Activity Index; CRP, C-reactive protein; CT, computed tomography; FC, fecal calprotectin; IV, intravenous; LOR, loss of response; MRI, magnetic resonance imaging.

**Figure 3.**
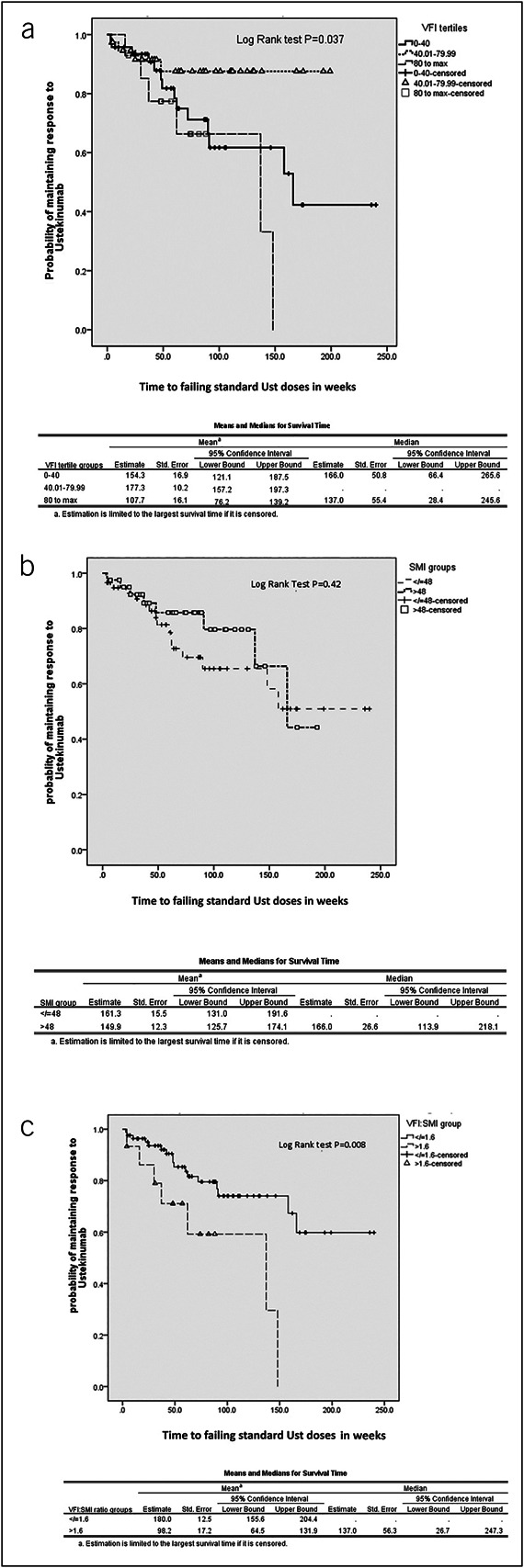
Kaplan-Meir survival analysis of time to failing ustekinumab (Ust) at standard doses in (**a**) visceral fat index (VFI) tertiles, (**b**) skeletal muscle index (SMI) group, and (**c**) VFI:SMI ratio group.

The cohort was analyzed by various BC parameters for time to ustekinumab failure at standard doses (Figure 3a–c). To demonstrate differences in survival times, the cohort was analyzed in tertiles or binomial groups depending on the range of measurements and the number of events in each group, to ensure events were evenly distributed. The midpoint in range of each BC parameter (skeletal muscle index [SMI], VFI:SMI ratio) or one-third points for VFI were used to derive the respective limits for comparing categories. Patients in the middle tertile category of VFI (40.01–79.99 cm^2^/m^2^) had the highest probability of maintaining response to standard doses of ustekinumab and significantly differed in time to ustekinumab failure at standard doses compared with patients in the lowest and highest tertile of VFI (Figure 3a). When individual subgroup comparisons (bottom 2 tertiles vs top tertile and top 2 tertiles vs bottom tertile) were performed for a sensitivity analysis, there was a signal for a trend in favor of the top tertile of VFI having the lowest survival probability of maintaining response to ustekinumab (mean survival 107.7 weeks vs 177.7 weeks, *P* = 0.06) (see Supplementary Figure 1b, Supplementary Digital Content 1, http://links.lww.com/CTG/B136). The lowest VFI tertile group having similarly inferior survival probabilities to the top VFI tertile group was further explored by correlating VFI with SMI, as these variables are often collinearly related (see Supplementary Figure 2, Supplementary Digital Content 2, http://links.lww.com/CTG/B137). There was a weak but statistically significant correlation between VFI and SMI (R = 0.31 *P* = 0.002), indicating that the lowest VFI tertile is likely to also have correspondingly low SMI. As SFA and AC were inaccurately measured as described in the Methods section, these variables were excluded from analysis. For similar reasons, total fat area could not be calculated.

SMI binomial groups using 48 cm^2^/m^2^ as the SMI range midpoint threshold did not show a significant difference with regard to time to failing standard ustekinumab doses (Figure 3b). The VFI:SMI ratio, however, using 1.6 as the range midpoint cutoff did show a signal for modulating time to failing standard ustekinumab doses, where the VFI:SMI >1.6 group, compared with the VFI:SMI ≤1.6 group, had a mean survival estimate of 98.2 weeks vs 180 weeks, respectively (*P* = 0.008) (Figure 3c).

Finally, univariate and multivariate Cox proportional hazard regression analyses were performed to determine associations with failing standard ustekinumab doses over time (Table 3). A younger age, smoking status (current vs ex-smoking/never smoking), and a higher VFI:SMI ratio notably increased the risk of meeting primary end point on the univariate analysis and met the criteria for being included in the multivariate model, where *P*-values were <0.2. After entering these covariates into the multivariate model, both a younger age at ustekinumab induction (hazard ratio [HR] 0.96, 95% CI 0.94–0.99, *P* = 0.01) and VFI:SMI ratio >1.6 (HR 4.65, 95% CI 1.73–12.45, *P* = 0.002) remained significant independent determinants for failing standard ustekinumab doses. BMI notably had no association with the primary outcome on both the univariate and multivariate analyses.

**Table 3.** Univariate and multivariate Cox proportional hazard analyses on risk of experiencing ustekinumab failure at standard doses

Co-variate	Univariate analysis			Multivariate analysis		
	HR	95% CI	*P* value	HR	95% CI	*P* value
Age, yr	0.98	0.95–1.00	0.11	0.96	0.94–0.99	0.01
Sex (ref = male)	1.6	0.71–3.6	0.26	—	—	—
BMI, kg/m^2^	1.01	0.95–1.08	0.67	—	—	—
Disease duration at induction, mo	1.00	0.99–1.00	0.48	—	—	—
Peak CRP (g/L)	1.00	0.99–1.00	0.67	—	—	—
Nadir albumin (g/L)	1.00	0.93–1.08	0.89	—	—	—
Concomitant IM >6 mo (ref = no)	1.58	0.67–3.62	0.29	—	—	—
Prior biologic failure (ref = no)	0.92	0.41–2.70	0.92	—	—	—
Presence of perianal disease (ref = P0)	1.8	0.71–4.50	0.23			
Smoking status: current vs never/ex	5.3	0.72–39.5	0.10	—	—	—
VFI (cm^2^/m^2^)				—	—	—
0–40.00 (ref)	1	—	—			
40.01–79.99	0.36	0.12–1.09	0.07			
≥80.00	1.71	0.65–4.55	0.28			
SMI (cm^2^/m^2^)				—	—	—
0–48.00	1	—	—			
≥48.00	0.71	0.30–1.60	0.42			
VFI:SMI ratio						
≤1.6	1	—	—	—	—	—
>1.6	3.2	1.29–7.84	0.01	4.65	1.73–12.45	0.002

BMI, body mass index; CI, confidence interval; CRP, C-reactive protein; HR, hazard ratio; IM, immunomodulator; SMI, skeletal muscle index; VFI, visceral fat index.

## DISCUSSION

To date, we have performed the largest study of the effect of BC on the long-term efficacy of ustekinumab. We found that of 99 patients who were induced with ustekinumab at standard doses, 24 inadequately responded to or eventually lost response to the biologic with a median time to reaching the primary end point of 166 weeks (95% CI 142.5–190.3). We found no association between BMI and ustekinumab failure at standard doses on univariate analysis, but the ratio of VFI to SMI strongly affected this outcome with a HR of 4.65 in those with a ratio >1.6, compared with the group with a ratio of ≤1.6 (*P* = 0.002) after multivariate adjustment. Age was also independently, albeit weakly and inversely associated with the risk of ustekinumab failure at standard doses (HR 0.96, 95% CI 0.94–0.99, *P* = 0.01). We did not find a statistically significant association of VFI and SMI individually on the primary outcome in the univariate analysis (albeit a trend to significance for VFI). The direction of the relationship, however, was in favor of a high VFI and a low SMI being associated with failing ustekinumab at standard doses.

IBD and obesity are both diseases of chronic inflammation with rising global prevalence pushing on an epidemiological and public health catastrophe. Fortunately, there is an increasing armamentarium of available agents that are effective in treating IBD. On the other hand, a significant proportion of patients inadequately respond to or lose response to consecutive advanced therapies, and there is an increasing interest in the phenotype of these patients, particularly those with a high BMI. Studies have diverged in their conclusions on the effect of BMI on response and remission status, particularly to anti-TNFα agents (2,3,6–9,14,15), with the weight of the data in favor of a high BMI having an inverse effect on clinical response and remission. In some studies, this finding is irrespective of whether weight-based or fixed SC doses were given (15,16). The divergent findings in studies using BMI as the covariate of interest have been limited by the fact that BMI is a crude measure of BC, with data linking biologic response with degree of intra-abdominal VAT% rather than BMI itself (6). There is limited but emerging data that this observation is also true of other biologics, including vedolizumab (3,15,17) and ustekinumab (3), but the data are sparse for the latter biologics. A recent *post hoc* analysis of the IM-UNITI maintenance trial found no association between BMI and clinical remission status despite an inverse association with ustekinumab trough level (10). Thus, we sought to evaluate this relationship further, by defining the role of BC on the risk of losing response to ustekinumab. Our findings corroborated with those of Wong et al, where BMI did not correlate with ustekinumab LOR; however, we found that a high visceral fat-to-skeletal muscle area ratio as measured by cross-sectional imaging did affect this outcome.

There are 2 major gains in understanding resulting from our data. First, we affirm that BMI is not a useful indicator of ustekinumab response after induction therapy, as depicted by Wong et al (10). Our previous work (9) and data from the PANTS study (8) showed that there was a null and inverse relationship for infliximab and adalimumab, respectively, regarding BMI and non-remission status. Thus, the lack of association of BMI with response to ustekinumab was unexpected, given that it is a fixed maintenance dose, despite a weight-stratified initial induction dose. These observations imply that the IV induction (weight-stratified) dose may have a greater impact on the maintenance of response than what is currently perceived. On the contrary, Kurnool et al found BMI to be a significant independent predictor for biologic failure, such that for every 1 kg/m^2^ increase in BMI, there was a 4% increased risk of failure, irrespective of mode of biologic delivery. However, the study examined patients with UC, whereas our study examined only patients with CD thus may have differed in results due to the slightly different biology of mesenteric adipose involvement between CD and UC.

Second, our results affirm those of Yarur et al who showed that intra-abdominal VAT % correlated well with steroid-free and endoscopic remission status of anti-TNFα agents, vedolizumab and ustekinumab (3). Until then, data on the effect of BC on ustekinumab-induced response or remission had not previously been reported. Their data, however, had a modest number of ustekinumab patients (n = 43) and included both patients with CD and UC (3). Nevertheless, Yarur et al were able to demonstrate that the relationship between BC and remission status did not appear to be related to ustekinumab trough levels as there appeared to be no correlation between trough levels and VAT%. We did not have enough ustekinumab trough levels available to analyze; however, we showed that the ratio of visceral fat to skeletal muscle area had an even stronger association to the primary outcome than VFI itself on both univariate and multivariate analyses, adding weight to the term “sarcopenic obesity.”

There is a high prevalence of concurrent obesity with sarcopenia seen in IBD patients with active and inactive disease (18,19). It has been shown that sarcopenic IBD patients have poorer clinical outcomes in terms of worse disease control, having a complicated disease course and a higher need for surgery (20–23). Our data add to this body of literature that viscerally fat and myopenic CD patients are at increased risk failing ustekinumab at standard doses. Whether the myopenia is the cause or effect of an inherently biologically resistant disease is unclear, and whether nutritional and exercise interventions to promote muscle hypertrophy would alter biologic resistance is also unknown. Further prospective studies are needed to answer the question if changing the BC of patients with IBD can recapture response, as has been shown affirmatively in the psoriasis literature (24).

There is a paucity of data on the change in BC over time in ustekinumab-treated IBD patients and whether it parallels the adiposity gains seen in anti-TNFα therapies (25). Two small studies in IBD (26,27) showed an increase in body weight with all biologics (anti-TNFα, vedolizumab, ustekinumab); however, ustekinumab was not associated with statistically significant weight gain compared with anti-TNFα and vedolizumab in one of these studies (27). Curiously, in patients with psoriasis, ustekinumab treatment have also led to insignificant weight gains, compared with anti-TNFα therapy (28). Taken together, with the limited data available, ustekinumab appears to be the favored biologic from an adipose gain point of view; however, further prospective studies with larger numbers comparing advanced therapies are required. Our data suggest that therapeutic resistance is seen in those of higher visceral fat mass relative to muscle mass and that the latter does have an effect on the longevity of ustekinumab efficacy. Until more data are available, increased awareness of weight management at the start of biologic therapy is needed to optimize biologic longevity, especially for those of a more rotund body phenotype.

Our study had several notable limitations. First, we had an inadequate number of subjects and primary outcome events to perform an AUROC analysis to determine the optimal BC cutoff level for maintaining ustekinumab response. For this reason, the midpoint in range of values for SMI and the VFI:SMI ratio were used to compare survival probabilities of remaining on standard doses of ustekinumab. The VFI range was divided and compared in thirds to differentiate the range in survival probabilities seen at the very high and very low ends of VFI values. Despite the small numbers, we were able to demonstrate a profound signal that warrants investigation with larger, preferably prospective studies. Second, as this was a retrospective study with all the inherent biases associated with such analyses, we had no data on the waist:hip ratio to compare the utility of this measurement with cross-sectional measurements of BC. Third, we could not use AC and SFA measurements in the analyses due to the inability to measure outside the field of view for our larger patients. For this same reason, we could not use total fat area. We used a mixture of CT and MRI scans thus could not uniformly measure intramuscular fat across all patients. Finally, we also lacked ustekinumab trough level measurements, as this was not routinely used in clinical practice at both centers.

The strengths of this study included first, the blinded design, where BC areas were measured by a single experienced radiologist using previously validated methods (9,13). Our study using radiological measurements has a pragmatic implication. Many of our patients with CD will have cross-sectional imaging at some stage, particularly before changing therapies and BC information can obtained from these scans. Second, despite the relatively small number of events, our cohort is still the largest to date that has examined the impact of BC on long-term ustekinumab outcomes, with important findings. Third, we used a stringent but pragmatic definition for the primary outcome, which made it more straightforward to objectively extract the data. Finally, our data were extracted from a prospectively maintained, combined operational and research database, where IBD nurses entered dates of induction and dose escalation on a real-time basis, giving us accurate treatment time lines of biologic failure.

In conclusion, a high ratio of visceral fat to skeletal muscle area measured at the L3 vertebral level on cross-sectional imaging is a strong determinant of failing standard doses of ustekinumab. Further prospective studies are needed to evaluate if changing a patient's BC toward a viscerally thinner and/or a more muscular body phenotype can prevent LOR, or recapture response, to ustekinumab.

## CONFLICTS OF INTEREST

**Guarantor of the article:** Lena Thin, MBBS, MClinRes.

**Specific author contributions:** L.T.: designed and conceived the study, perform data analysis, contributed to initial and finalization of the final draft. Z.T.: collection of data, contributed to initial draft. A.C.: collection of data. C.J.W.: contributed to data collection, contributed to initial and final draft. All authors have seen the final version of the article and have given their approval for publication.

**Financial support:** None to report.

**Potential competing interests:** L.T. receives advisory board/steering committees fees for education from AbbVie, Takeda, Janssen, Ferring, BMS, Pfizer, Celltrion, Chiesi; receives research grants from Celltrion, Takeda, Pfizer; and is an executive member of the Australian New Zealand IBD consortium. The other authors have no conflicts of interest to disclose.

**Data availability statement:** Data is available on request and approval of the South Metropolitan Area Health Service Human Research Ethics Committee.

**Study Highlights**

**WHAT IS KNOWN**

✓ There is increasing evidence linking body composition with disease activity in patients with inflammatory bowel disease and response to biologic therapies, mostly in anti-tumor necrosis factor α literature. There is a paucity of data on the relationship between body composition and ustekinumab therapeutic response.

**WHAT IS NEW HERE**

✓ Our multicenter study found that a high ratio of visceral fat to skeletal muscle area measured at the L3 vertebral level on cross-sectional imaging is a strong determinant of failing to respond or losing response to standard doses of ustekinumab.

## Supplementary Material
