# Supplementary material for: A High Visceral-to-Skeletal Muscle Area Ratio on Cross-Sectional Imaging Is Associated With Failure of Standard Ustekinumab Doses: A Multicenter Study
Source: Clin Transl Gastroenterol. 2024 Jun 1;15(7):e00722. doi: 10.14309/ctg.0000000000000722 (PMC11272374; doi:10.14309/ctg.0000000000000722)
Supplement: Supplementary file 1 [file ct9-15-e00722-s001.docx]

**Supplementary Figure 1**

**Figure 1a Figure 1b**


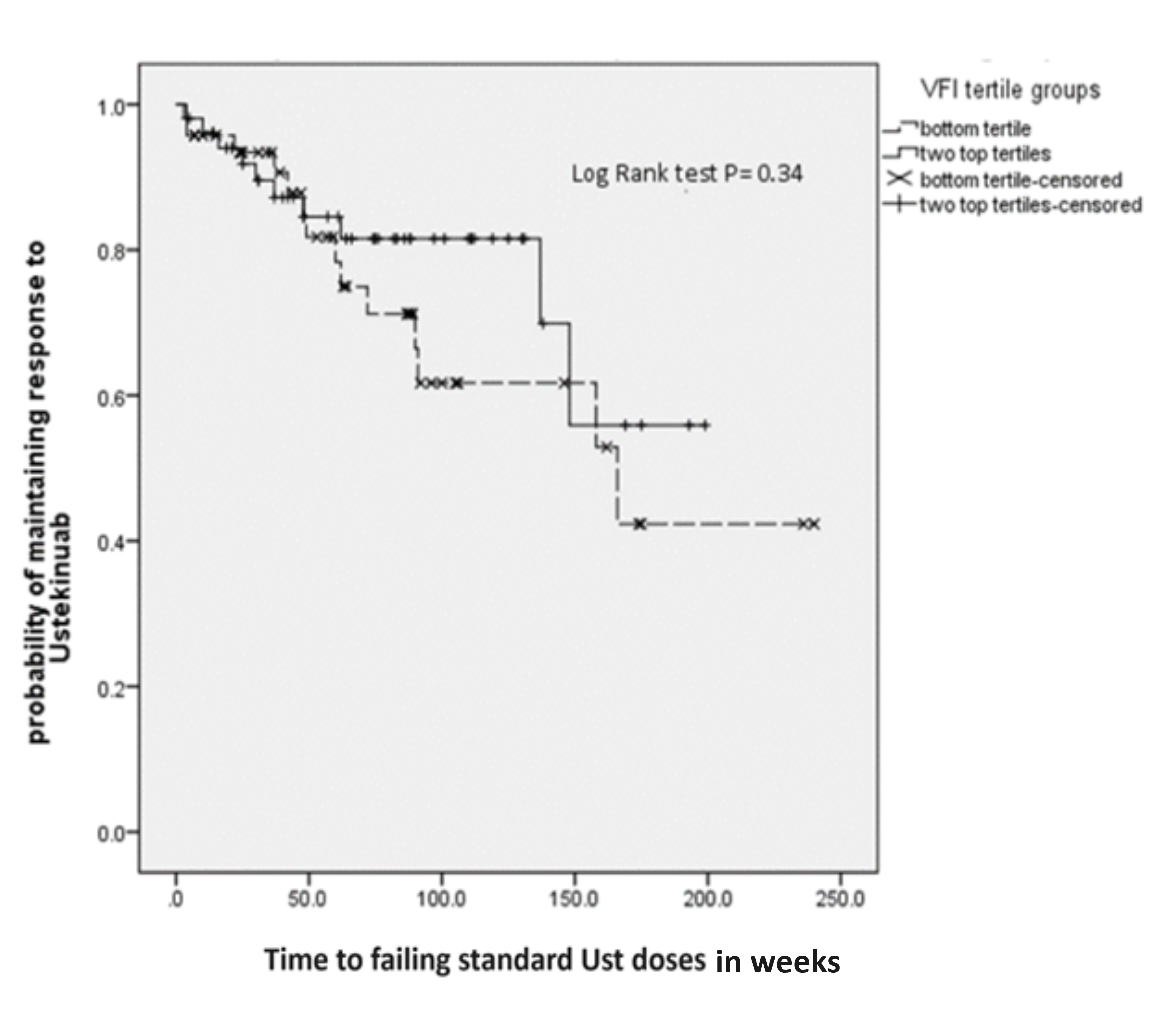

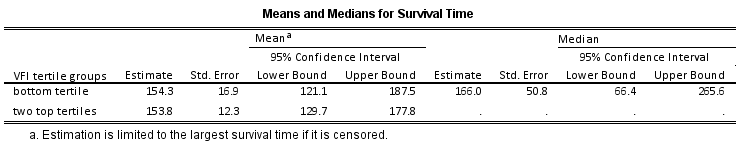


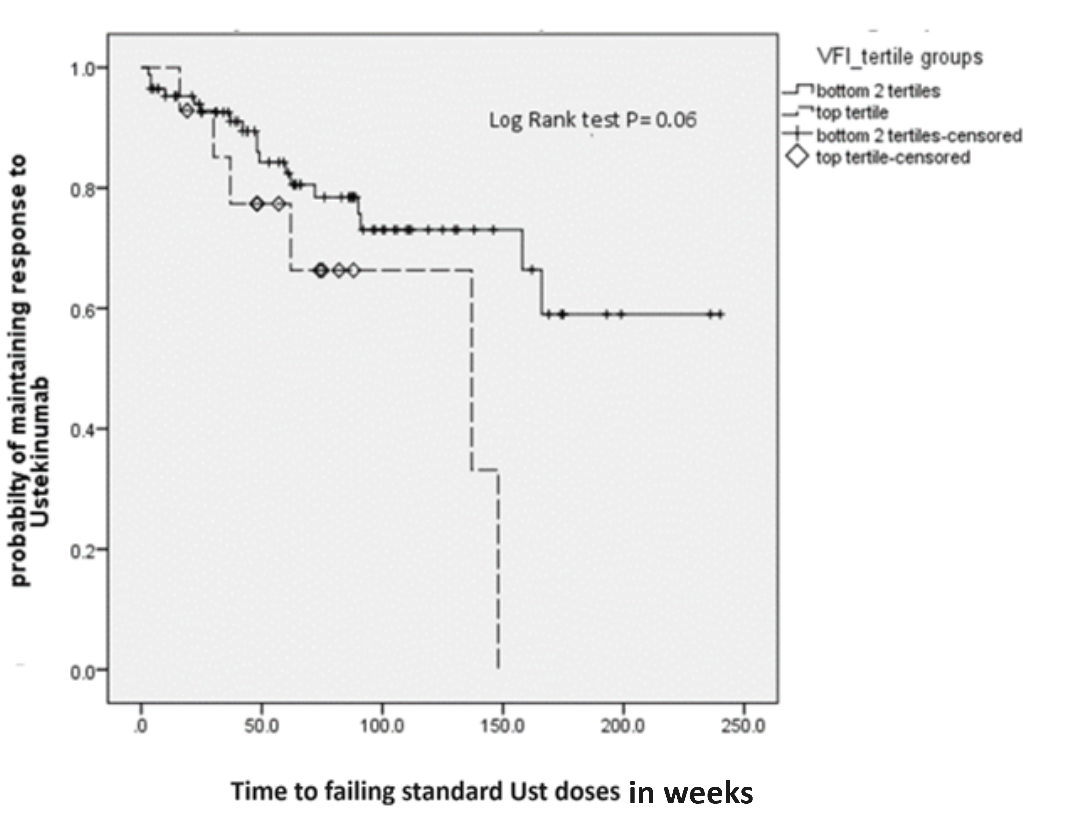


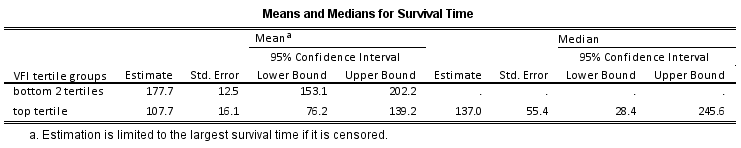


Figure 1a depicts the difference in time to failing standard Ust doses in weeks of the bottom VFI tertile compared to the top two VFI tertiles and Figure 1b depicts the difference in time to failing standard Ust doses in weeks of the bottom two VFI tertiles compared to the top VFI tertile.
