# Supplementary figures and images for: A High Visceral-to-Skeletal Muscle Area Ratio on Cross-Sectional Imaging Is Associated With Failure of Standard Ustekinumab Doses: A Multicenter Study
Source: Clin Transl Gastroenterol. 2024 Jun 1;15(7):e00722. doi: 10.14309/ctg.0000000000000722 (PMC11272374; doi:10.14309/ctg.0000000000000722)

**Supplementary Figure 2**

Correlation of skeletal muscle index (SMI) with visceral fat index (VFI).

**
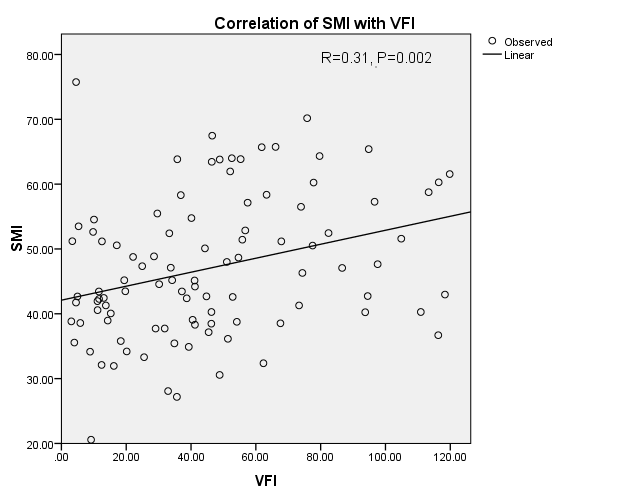
**

Supplement: Supplementary file 2 [file ct9-15-e00722-s002.docx]
